# Spatiotemporal analysis and forecasting of public attention to China’s five major religions

**DOI:** 10.1038/s41598-025-15396-9

**Published:** 2025-08-08

**Authors:** Xianhang Xu, Mohd Anuar Arshad, Hong Liu, Mengjiao Zhao, Jiejing Yang, Shuxia Cao, Guoyu Luo, Ming Chen, Qianqian Chen

**Affiliations:** 1 School of Management, Chongqing Institute of Engineering, Chongqing, 400056 China; 2 School of Management, Universiti Sains Malaysia, Penang, 11800 Malaysia; 3 School of Economics and Management, Tianjin Tianshi College, Tianjin, 301700 China; 4 School of Business, Changshu Institute of Technology, Suzhou, 250000 China; 5 School of Business, Henan Kaifeng College of Science Technology and Communication, Kaifeng, 475004 China

**Keywords:** Public attention, Religion, Spatiotemporal analysis, GIS technology, Forecasting, Mathematics and computing, Applied mathematics, Computational science, Scientific data, Statistics

## Abstract

In the digital era, leveraging search engine data to gain insights into public interests and attitudes towards religion has become increasingly important. To examine the spatiotemporal evolution and develop a forecasting model of public attention to China’s five major religions (Buddhism, Taoism, Catholicism, Christianity and Islam), this study introduces geographic information system technology, forecasting technology and spatiotemporal analysis methods into religious research, using Baidu Index data from 2020 to 2024. The results show that most religions exhibit stable annual public attention, although significant changes occur during specific seasons, festivals or events. Spatial variation in public attention is moderate, with a relatively balanced regional distribution. However, a distinct east–west clustering pattern is evident, reflecting spatial aggregation. The SARIMA-based forecasting model effectively captures temporal dynamics and demonstrates strong forecasting performance. Findings reveal the spatiotemporal distribution patterns of religious public attention and highlight the practical value of the forecasting model, thereby emphasising the importance of considering spatial factors and forecasting models when addressing the dissemination of religion in the digital age. This study provides new methods and perspectives for religious research, promoting an interdisciplinary synthesis of religious studies, sociology and geography. It offers new insights into global religious studies, religious communication strategies and cultural exchange. Furthermore, it contributes to advancing cross-cultural and cross-regional religious research and offers valuable references for religious organisations and policymakers in managing religious affairs, adjusting policies and optimising communication strategies in a globalised context.

**Supplementary Information:**

The online version contains supplementary material available at 10.1038/s41598-025-15396-9.

## Introduction

The internet has become deeply embedded in the global economy and everyday life. According to the *Digital 2025: Global Overview Report*, at the beginning of 2025, the number of global internet users is 5.56 billion, making up 67.9% of the population. Furthermore, Google is the most visited website in the world, and search engines are still the main way people learn about brands^1^. Because search engines record a large number of user searches and interests^2,3^ they are useful tools for tracking changes in public attention^4^.

Religion is a complex phenomenon of society, closely tied to people’s emotions, beliefs, and values^5^. It plays an important and far-reaching role in social life^6^. In China, the major religions followed by believers are Buddhism, Taoism, Catholicism, Christianity (specifically referring to Protestantism) and Islam. These are commonly known as the five major religions in China^7^. Buddhism originated in ancient India and entered China around the 1 st century AD. It gradually merged with Han Chinese and ethnic minority cultures, forming three main branches: Chinese Buddhism (Han tradition), Tibetan Buddhism, and Theravada Buddhism in Yunnan^8^. Taoism emerged in the 2nd century AD as a major religion that originated and developed within China^9^. Founded by Laozi and based on the *Tao Te Ching*, it is deeply rooted in Chinese philosophy and spiritual life^10^. Christianity originated in West Asia and experienced three waves of introduction and interruption in China. In the modern era, Catholicism and Protestantism underwent reforms of indigenisation and self-governance during national reform movements, establishing independent and self-managed religious communities^11^. Islam was founded by the Prophet Muhammad in the 7th century on the Arabian Peninsula and entered China during the Tang and Song dynasties via Arab merchants. Over time, it gradually integrated into local society and became an organic part of China’s multi-ethnic national framework^12^.

These five religions are officially recognised by the Chinese government and represent the main spiritual traditions in the country. They have a long history in China and have developed various branches with significant scale and regional influence^13^. Widely accepted by religious communities across the country^14^ they provide spiritual support for many people and contribute to social stability and cultural continuity^15^. Therefore, they are included in this study to reflect the diverse and complex nature of religion in China.

China’s five major religions have played an important role in the country’s history and cultural growth^16^. In today’s digital age, the internet has become a major way for people to learn about religion and express their faith. Public interest in religion also reflects how society views different beliefs^17^. Each religion in China has its own history and way of blending with local culture. Religious activities differ from place to place, influenced by festivals, worship habits, geography, and local traditions^18,19^. As a result, public attention to these religions changes over time and varies across regions.

Nonetheless, relatively few studies have examined the spatiotemporal changes in public interest in religion. Analysing these changes can enhance our understanding of religion’s role in society and inform religious governance and policymaking. With the advancement of big data tools, geographic information systems (GIS), and time series forecasting models, such research has become increasingly feasible. Online platforms like Google Trends and Baidu Index offer valuable data on public interest. Forecasting models reveal temporal trends, while GIS helps visualize spatial patterns. Compared to traditional methods, these approaches provide more accurate results and stronger scientific support.

In general, this study aims to investigate the spatiotemporal distribution patterns of public attention toward China’s five major religions and develops a forecasting model. The research objectives (ROs) are as follows:

RO1: To analyse the temporal characteristics of public attention by annual and quarterly data, revealing long-term trends and seasonal variations.

RO2: To assess the spatial evolution of public attention and visualise the patterns through GIS technology for cross-regional comparison.

RO3: To construct and validate a forecasting model for public religious attention using the Seasonal Autoregressive Integrated Moving Average (SARIMA) approach.

To address these objectives, this study employs public attention data encompassing all 31 regions of mainland China from 2020 to 2024. A combination of GIS technology, spatiotemporal indicators, spatial autocorrelation analysis, and SARIMA modelling is employed. The analytical process is carried out using Excel, GeoDa, and Python software.

Based on the research method and objectives, this study will address the following research questions (RQs).

RQ1: What are the temporal characteristics of public attention to China’s five major religions over different periods (yearly and quarterly)?

RQ2: What are the spatial distribution patterns of public attention to China’s five major religions across different regions in China?

RQ3: Can the SARIMA model effectively forecast the public attention to China’s five major religions, and how accurate is the model’s performance?

This study applies GIS technology, forecasting technology and spatiotemporal analysis methods to religious studies and offers novel ideas and methods for quantitative research into religious issues. It promotes interdisciplinary research across religious studies, sociology, geography and other fields, which has theoretical significance; by analysing the spatiotemporal evolution and forecasting model of public attention to China’s five major religions, this study provides a deeper understanding of people’s religious demands in the internet era. It offers relevant organisations data and decision support for to manage religious affairs better and leverage religion’s positive roles, which has practical significance; It employs diverse analytical approaches that are applicable across religious studies and other fields within the social sciences, thereby offering wider methodological contributions.

## Literature review

Search engine data reflect the active search behaviour of internet users. Scholars have utilised it to study people’s lives and economic activities, including public health^20–23^ culture^23–27^ economics and finance^28–32^ education^33^ and business activities^34–36^. The forecasting methods used in these studies include multiple regression analysis^37–39^ time series analysis^40,41^ machine learning^42,43^ and deep learning^44–47^. Search engine data analysis has emerged as a powerful methodological approach for monitoring and forecasting public behaviour, representing a growing area of research interest.

Recent studies have increasingly leveraged digital trace data, particularly from platforms like Google Trends, to examine religious behaviour. These studies illuminate the complex interactions among religious belief systems, cultural norms, geographical factors, and digital engagement. For instance, Adamczyk et al.^48^ demonstrated how regions with strong religious conservatism exhibit both intensified interest in and resistance to non-normative behaviours. Wormley and Cohen^49^ found that states with higher levels of religiosity reported fewer instances of cheating in online games. Uğur and Özdemir^50^ observed a notable decline in pornography-related searches during Ramadan, particularly in Turkey’s more devout provinces, while Mnif et al.^51^ demonstrated that religious belief significantly influences users’ willingness to engage with cryptocurrencies.

These studies underscore the interaction between religiosity and individual behaviour, shaped by both temporal and spatial factors. Moreover, other research has highlighted the connection between religious beliefs and broader cultural dimensions, as well as the methodological reliability of digital tools in religious research. Perry and Whitehead^52^ identified a cultural link between religiosity and masculinity through regional search data. Kayal^53^ found that self-esteem and relational needs influence individuals’ intentions to visit religious sites. Tan et al.^54^ emphasised the role of Buddhism in alleviating economic stress related to well-being in China. Adamczyk et al.^55^ further confirmed the utility of Google Trends in capturing cross-national differences in religious attention.

Together, these studies reflect the growing scholarly interest in using digital data to explore religion as a sociocultural phenomenon, and in highlighting variations in belief and behaviour across different temporal and geographical contexts. The origins and development of religion are closely linked to environmental and cultural conditions^56^. Regional religious participation and behaviour are often shaped by underlying belief systems and sociohistorical backgrounds. Accurately identifying the spatiotemporal characteristics of religious attention is therefore essential for understanding religious diffusion^57^. Exploring the spatial and temporal distribution and forecasting trends in public attention not only enriches sociological insight into religion but also provides valuable guidance for the governance and management of religious affairs.

Geographic Information System (GIS) technology serves as a foundation of this research, providing powerful spatial analysis capabilities that enable novel analytical perspectives and methodological approaches. Since its inception in the 1960 s, GIS technology has made significant advancements and gained widespread interdisciplinary adoption^58^. Recent studies have demonstrated increasing interest in coupling GIS with search engine analytics across multiple domains, including tourism^59–61^ public health^62,63^ healthcare^64,65^ agriculture^66^ and social science^67,68^. The integration of big data platforms (e.g., Google Trends, Baidu Index) with GIS enables researchers to perform high-resolution spatial analyses and generate dynamic visualizations that elucidate complex spatiotemporal patterns^69,70^. Furthermore, GIS facilitates advanced spatial autocorrelation analysis^71^ providing critical insights on the interaction between religious dissemination dynamics and the broader social environment.

Existing research on public attention has yielded valuable insights for scientifically analysing religious interest and its societal impacts. However, current studies frequently examine isolated aspects of religion, such as individual behaviours or attitudes, while neglecting systematic investigation of spatial patterns and evolutionary trends in religious belief and influence. The spatial distribution of religion both reflects and shapes demographic characteristics, while being deeply embedded in religious doctrines, cultural traditions, and socio-political dynamics. By leveraging search engine data to analyse the spatiotemporal patterns of public attention in religious, we can achieve a ‌profound understanding of the broader process of religious diffusion, interactions and evolution in social change. In addition, using models to forecast public attention can also help grasp the macro trends of religious and cultural changes.

This study integrates GIS technology and forecasting technology to religious studies, offering new perspectives and methods, revealing complex distribution spatiotemporal patterns and providing scientific evidence for the government to formulate religious policies and optimise resource allocation. Moreover, this approach can promote the development of interdisciplinary research and expand the application of these technologies in social sciences.

## Data and method

### Sample and data source

This study selected internet users from 31 provinces in mainland China as samples and used their public attention data for analysis, which is reflected in the Baidu Index. The Baidu Index was selected because Baidu is the most used Chinese search engine^72^. It computes the weighted search frequency of each keyword in Baidu searches based on certain rules^73^ reflecting the searching behaviour of netizens on focal events and related topics^74^. Although Baidu Index data may not be extremely precise due to retrieval sampling and approximation algorithms, it can provide insights into user attention trends^75^. These data have been used widely as a reference for public attention and are considered representative and authoritative.

This study used Baidu Index data from 2020 to 2024, focusing on five keywords that represent the major religions in China: Buddhism (佛教), Taoism (道教), Catholicism (天主教), Christianity (基督教), and Islam (伊斯兰教). These keywords are widely used and officially recognized, and all can be tracked through the Baidu Index. To keep the analysis clear and easy to manage, one keyword was chosen for each religion. This helps keep the categories balanced and avoids unfair comparisons. Using a single keyword also reduces confusion and prevents errors that might come from combining several search terms. It allows for a more direct and accurate view of public interest in each religion. This method also makes it easier to compare the five religions and ensures that the results can be repeated in future studies. The selected keywords are commonly used by Baidu users, which helps make the data more reliable and representative.

### Research method

This study is quantitative and the data analysis used include Excel, Python and GeoDa. First, the spatiotemporal evolution of public attention was examined using a range of spatiotemporal indicators, such as the annual variation index (AVI), seasonal intensity index (SII), coefficient of variation (CV) and Moran’s I. Meanwhile, an analysis of variance (ANOVA) was used to verify the results. In addition, to visually explore the local spatial autocorrelation of public attention, local spatial correlation maps were generated. Finally, a forecasting model was constructed and its accuracy was subsequently evaluated.

#### Spatiotemporal indicators

(1) AVI reflects the year-on-year changes in public attention^76^. The equation is as follows:

1 $$\:AVI=\frac{{X}_{i}}{\frac{1}{n}\sum\:_{i=1}^{n}{X}_{i}}$$

Here, *Xi* represents the public attention in year *i*. The further the AVI value deviates from 1, the greater the interannual variation in public attention, indicating a higher level of instability.

(2) SII indicates the degree of temporal concentration in public attention, reflecting whether public attention is seasonally concentrated or evenly distributed over time^77^. The equation is as follows:

2 $$\:SII=\sqrt{\sum\:_{i=1}^{12}\frac{{\left({X}_{i}-8.33\right)}^{2}}{12}}$$

Here, *Xi* represents the relative value of monthly public attention in relation to the annual total. A higher SII value indicates greater seasonal variation and a more concentrated monthly distribution of public attention. Conversely, an SII value approaching 0 suggests a more uniform distribution across months.

(3) CV reflects the degree of regional disparity in public attention across multiple areas^78^. The equation is as follows:

3 $$\:CV=\sqrt{\sum\:_{i=1}^{n}{\left({X}_{i}-\stackrel{-}{X}\right)}^{2}/n}/\stackrel{-}{X}$$

Here, *X*_*i*_ denotes the public attention in region *i*, and *n* represents the number of local administrative units (in this study, *n* = 31). A higher CV value indicates greater spatial variation in public attention. Specifically, CV > 1 suggests substantial variation, 0.1 < CV ≤ 1 indicates moderate variation, and CV ≤ 0.1 reflects weak variation.

(4) Spatial autocorrelation analysis.

Spatial autocorrelation analysis is a commonly used spatial econometric technique^79^ including global or local based on the content being examined^80^. This study applies both to investigate the spatial correlation of public attention. The equations are as follows:

4 $$\:I=\frac{n}{\sum\:_{i=1}^{n}\sum\:_{j=1}^{n}{W}_{ij}}\times\:\frac{\sum\:_{i=1}^{n}\sum\:_{j=1}^{n}{W}_{ij}\left({X}_{i}-\stackrel{-}{X}\right)\left({X}_{j}-\stackrel{-}{X}\right)}{\sum\:_{i=1}^{n}{\left({X}_{i}-\stackrel{-}{X}\right)}^{2}}$$

5 $$\:{I}_{L}=\frac{n}{\sum\:_{i=1}^{n}\sum\:_{j=1}^{n}{W}_{ij}}\times\:\frac{\left({X}_{i}-\stackrel{-}{X}\right)\sum\:_{j=1}^{n}{W}_{ij}\left({X}_{j}-\stackrel{-}{X}\right)}{\sum\:_{j=1}^{n}{\left({X}_{i}-\stackrel{-}{X}\right)}^{2}}$$

Here, *I* and *I*_*L*_ represent the Global and Local Moran’s I indices, respectively; *X*_*i*_ and *X*_*j*_ denote the public attention values for regions *i* and *j*; *W*_*ij*_ is the spatial weight; and *n* is the total number of regions under examination. If there is a geographical border between regions *i* and *j*, *W*_*ij*_ is assigned a value of 1; otherwise, it is set to 0.

A value of *I* = 0 indicates a random spatial distribution of the observed variable. A positive value (*I* > 0) suggests a positive spatial correlation, implying a clustering pattern, whereas a negative value (*I* < 0) indicates a negative spatial correlation, reflecting a dispersed or heterogeneous spatial distribution^81^. To verify the significance of *I*, the Z-score is commonly used, thereby ensuring the statistical robustness and reliability of the results. The equation is as follows:

6 $$\:Z\left(I\right)=\frac{\left[I-E\left(I\right)\right]}{\sqrt{VAR\left(I\right)}}$$

Here, *Z(I)* denotes the significance level of Moran’s *I*, *E(I)* its expected value, and *VAR(I)* its variance. If |*Z*|>1.96, spatial autocorrelation is significant; otherwise, the distribution is random^82^.

Local spatial autocorrelation identifies spatial patterns such as clustering locations, reflecting the relationship between observed values and those of neighbouring units within a given spatial context^83^. According to the *I*_*L*_, spatial clusters can be classified as high–high (H–H), low–low (L–L), high–low (H–L), and low–high (L–H). H–H and L–L indicate spatial correlation in public attention, while H–L and L–H reflect spatial heterogeneity.

#### Forecasting model

Given the objective of forecasting temporal patterns in public religious attention with high interpretability, this study adopted the SARIMA model. SARIMA is particularly effective for short-term, seasonal time series data with relatively limited sample sizes. Compared to machine learning models, SARIMA offers greater transparency and parameter interpretability, making it less prone to overfitting and more suitable for exploratory research where explainability is critical. These advantages make it an appropriate choice for modelling the monthly Baidu Index data across the five major religions.

(1) SARIMA model

Autoregressive Integrated Moving Average (ARIMA) is a classical time series method. The model integrates autoregressive (AR) and moving average (MA) components while utilising differencing to satisfy stationarity conditions. ARIMA model has been widely applied in various fields. SARIMA is an extension of ARIMA, incorporates parameters related to data seasonality, making it more suitable for forecasting time series with significant seasonal patterns and large data volumes^40^. Consequently, SARIMA proves particularly effective for modelling public attention trends. This study applies the SARIMA approach to forecast public attention to China’s five major religions.

The SARIMA model follows the notation SARIMA (*p*,* d*,* q*) × (*P*,* D*,* Q*)_s_, with *p*, *d*, and *q* corresponding to the non-seasonal autoregression, differencing, and moving average terms in that order. *P*, *D*, and *Q* represent the orders of the seasonal autoregressive, differencing, and moving average components with a seasonal period of *s*^84^. The equation is as follows:

7 $$\:{\Phi\:}_{P}\left({B}^{s}\right)\phi\:\left(B\right){\left(1-{B}^{s}\right)}^{D}{\left(1-B\right)}^{d}{y}_{t}=c+{\varTheta\:}_{Q}\left({B}^{s}\right){\theta\:}_{q}\left(B\right){\varepsilon}_{t}$$

Here, *y*_*t*_ represents the value of the time series at time *t*, and *ε*_*t*_ denotes the white noise term at time *t*; *c* is a constant term; *B* is the backshift (lag) operator; *B*^*s*^ indicates a shift of the time series by *s* periods, i.e., *B*^*s*^*y*_*t*_=*y*_*t*−*s*_; *ϕ*_*p*_(*B*) and *θ*_*q*_(*B*) represent the non-seasonal autoregressive and moving average polynomials of orders *p* and *q*, respectively; *Φ*_*P*_(*B*^*s*^) and *Θ*_*Q*_(*B*^*s*^) denote the seasonal autoregressive and moving average polynomials of orders *P* and *Q* with seasonal period *s*, respectively.

Thus, a SARIMA model is characterised by seven parameters: *p*, *d*, *q*, *s*, *P*, *D*, and *Q*. The process of determining these parameters is referred to as model identification^85^.

(2) Data testing

Stationarity refers to the consistent statistical characteristics of a time series over a period, without exhibiting trends or seasonality. In other words, the mean and variance remain stable throughout the series^86^. Stationarity can be assessed using unit root tests, such as the Augmented Dickey-Fuller (ADF) test^87^. If a time series is non-stationary, differencing is applied to transform it into a stationary series, satisfying the assumptions of autoregressive and moving average models.

The non-white noise test evaluates the presence of autocorrelation within the series, typically using the Ljung–Box Q test^88^. A non-white noise result suggests that current observations influence future values, indicating the series is forecastable. Conversely, if the series is white noise, it is considered random and unpredictable. In this study, the first-order differenced public attention series was found to be non-white noise, confirming that it can be effectively forecasted based on its inherent structure.

(3) Performance metrics

The accuracy of forecasting results largely depends on the estimation and comparison between actual and forecasted values. Commonly used accuracy metrics include Mean Absolute Percentage Error (MAPE), Root Mean Squared Error (RMSE), and Directional Accuracy (DA). These performance indicators are defined as follows^89^:

8 $$\:MAPE=\frac{1}{n}{\sum\:}_{i=1}^{n}\left|\frac{{y}_{i}-{y}_{i}^{{\prime\:}}}{{y}_{i}}\right|$$

9 $$\:RMSE=\sqrt{\frac{1}{n}{\sum\:}_{i=1}^{n}{\left({y}_{i}-{y}_{i}^{{\prime\:}}\right)}^{2}}$$

10 $$\:DA=\frac{1}{n}{\sum\:}_{i=1}^{n}{a}_{i}$$

Where,

$$\:{a}_{i}=\left\{\begin{array}{c}1,({y}_{i}-{y}_{i-1})({y}_{i}^{{\prime\:}}-{y}_{i-1}^{{\prime\:}})\ge\:0\\\:0,({y}_{i}-{y}_{i-1})({y}_{i}^{{\prime\:}}-{y}_{i-1}^{{\prime\:}})<0\end{array}\right.$$

Here, *n* denotes the number of samples; *a*_*i*_ indicates whether the predicted and actual values move in the same direction at time *i*, *yi* and $y_{i}{}^\prime$ represent the actual and forecasted values of the *i*-th sample, respectively; $y_{i-1}$ and $y^\prime_{i-1}$ represent the actual and forecasted values of the (*i*–1)-th sample, respectively.

Lower values of MAPE and RMSE indicate better forecasting performance. The value of DA ranges from 0 to 1, with values closer to 1 indicating that the forecasted direction of change more closely aligns with the actual trend.

**Fig. 1:**
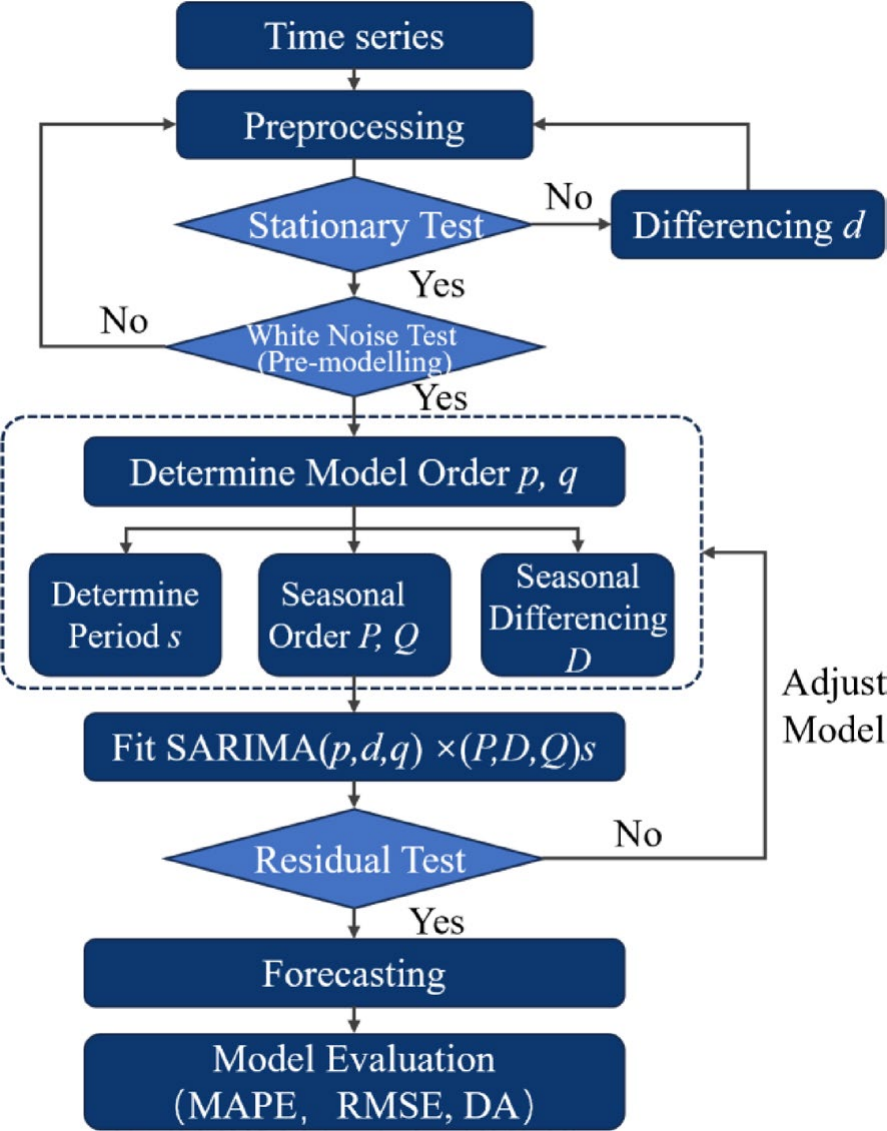
The methodology of the SARIMA model.

Figure 1 presents the methodological framework of the SARIMA model, which comprises the following key steps: data stabilisation, model identification and fitting, residual testing, forecasting, and evaluation.

## Results

### Time characteristics

#### General trend

**Fig. 2:**
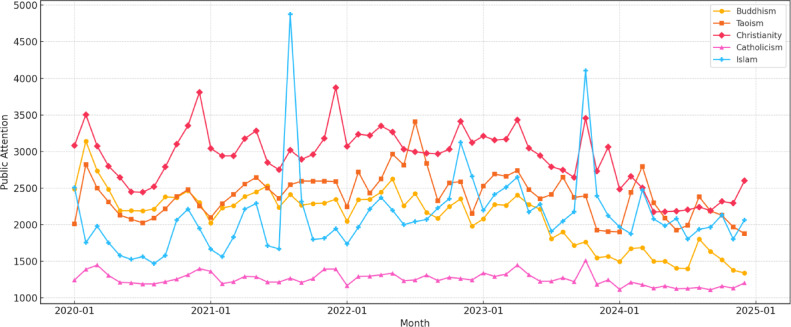
The changing trend of public attention to China’s five major religions (2020–2024).

According to Fig. 2, the changing trends in public attention to China’s five major religions from 2020 to 2024 reveal the following patterns: (1) Buddhism shows a clear downward trend, indicating a decline in its online appeal—possibly due to shifting public interests or the emergence of alternative cultural or religious influences. Taoism and Christianity exhibit an initial rise followed by a decline, reflecting cyclical fluctuations likely influenced by religious festivals, cultural events, or social dynamics. Catholicism remains relatively stable with slight fluctuations, but consistently low attention suggests limited online visibility within the Chinese digital sphere. Islam demonstrates a general upward trend, potentially driven by increasing interest in socio-political topics or Islamic culture and religious knowledge. (2) In horizontal comparison, Christianity receives the highest public attention, while Catholicism remains the lowest—possibly reflecting Christianity’s more developed online communication strategies. In terms of monthly variations, Buddhism and Taoism remain stable throughout the year. Catholicism and Christianity peak in December, likely due to Christmas. Public attention to Islam is generally stable, with occasional fluctuations. Two notable spikes were observed in August 2021 and October 2023. The rise in August 2021 may be linked to increased domestic media coverage about Islamic practices and religious freedom in Xinjiang^90^ which likely drew public attention and boosted online searches. The October 2023 spike happened during the Israel–Hamas conflict^91^ which was widely reported in Chinese media. Since this conflict is closely tied to Islam, it may have led to more online discussions and search activity. These patterns suggest that both domestic political topics and major international religious events can strongly affect public interest in religion in China.

Overall, the fluctuations in public attention are relatively limited except specific festivals or events, suggesting that while socio-cultural and political factors influence online interest, the digital visibility of religion remains relatively stable. These trends are essential for understanding how religion engages with the public in the digital era. Religious organisations and scholars should prioritise effective online communication strategies and enhance the appeal of digital content to sustain or increase public attention. Moreover, seasonal and festival-related factors should be considered when designing religious activities and content.

#### Annual variation index (AVI)

Applying Eq. (1), the annual variation in public attention to China’s five major religions was measured. As shown in Table 1, the AVI values for Buddhism range from 0.7258 to 1.1524, with a maximum deviation of 0.4266, suggesting the highest level of annual instability. Christianity and Islam also exhibit considerable variability, with maximum deviations of 0.2757 and 0.2754, respectively, suggesting relatively unstable public attention. By contrast, Taoism shows greater stability, with a maximum deviation of 0.1977, indicating limited annual variation. Catholicism demonstrates the highest level of stability, with the lowest maximum deviation (0.1202), implying minimal year-on-year fluctuation. Overall, Buddhism, Christianity, and Islam exhibit relatively high annual variability, while Taoism and Catholicism maintain more stable levels of public attention over time.

**Table 1:** AVI of public attention to china’s five major religions (2020–2024).

Year	Buddhism	Taoism	Catholicism	Christianity	Islam
2020	1.1524	0.9481	1.0204	1.0181	0.8584
2021	1.0978	1.0353	1.0172	1.0580	1.0076
2022	1.0811	1.1012	1.0100	1.0786	1.0545
2023	0.9429	1.0119	1.0363	1.0425	1.1338
2024	0.7258	0.9035	0.9161	0.8029	0.9457
Max Deviation	0.4266	0.1977	0.1202	0.2757	0.2754

An ANOVA was performed on the AVI values across the five major religions, and the results showed an F-value of 3.26 × 10⁻⁸ with a P-value close to 1. The extremely low F-value and the P-value well above the significance threshold of 0.05 indicate that there is no significant statistical difference in AVI between different religions. This suggests that the observed annual variations in public attention are likely attributable to random fluctuations rather than systematic differences between religions. Interannual variability in public attention appears to be relatively consistent across all five religions, with none demonstrating significantly greater or lesser stability than the others. For researchers and policymakers, this implies that these religions may employ similar digital engagement strategies, as their patterns of public attention display comparable temporal behaviours.

#### Seasonal intensity index (SII)

Equation (2) was used to assess seasonal variation in public attention. As shown in Table 2, Islam exhibits the strongest seasonality (mean SII = 1.7253), with a sharp increase in 2021, likely linked to observances such as Ramadan. Catholicism shows the weakest seasonality (mean SII = 0.4252), with a clear downward trend. Buddhism (0.7941) and Taoism (0.8632) demonstrate moderate seasonality with fluctuating trends, while Christianity (0.7272) shows a declining seasonal pattern. The seasonal peaks for Christianity and Catholicism likely correspond to Christmas, though Catholicism’s influence is diminishing. In summary, Islam is most affected by seasonal factors, Catholicism the least, and the remaining religions display moderate seasonal variation.

**Table 2:** SII of public attention to china’s five major religions (2020–2024).

Year	Buddhism	Taoism	Catholicism	Christianity	Islam
2020	0.9120	0.8428	0.5554	1.1750	1.3986
2021	0.4471	0.5244	0.4491	0.7597	3.3458
2022	0.6471	1.0459	0.2915	0.3880	1.2878
2023	1.2170	0.8956	0.5941	0.7063	1.8834
2024	0.7471	1.0075	0.2358	0.6070	0.7107
Mean	0.7941	0.8632	0.4252	0.7272	1.7253

ANOVA results for SII showed significant differences in seasonal variation among the religions (F = 4.8233, *p* = 0.0069). These differences suggest that certain religions experience more pronounced seasonal fluctuations in public attention, likely influenced by religious festivals, cultural practices, or annual events. Understanding these patterns can help religious organisations optimise their online engagement strategies—for example, by targeting activities during peak periods in high-seasonality religions. This finding highlights the importance of incorporating temporal dynamics when analysing religious behaviour in the digital context.

### Spatial characteristics

#### Coefficient of variation (CV)

Equation (3) was applied to assess regional variation in public attention to China’s five major religions. As shown in Table 3, the CV values across all five religions range from 0.3 to 0.5, indicating a moderate degree of spatial variation that remains relatively stable over the years. This moderate variability likely reflects differences in regional culture, religious practices, and demographic composition. Among the five, Christianity exhibits the highest mean CV value (0.4181), suggesting greater regional disparity in public attention—possibly due to uneven distribution of Christian communities or varying levels of religious activity. In contrast, Islam had the lowest mean CV (0.3600), indicating a more consistent public attention across regions. This may be attributed to the geographical concentration of the Muslim population in regions such as Ningxia and Xinjiang.

**Table 3:** CV of public attention to china’s five major religions (2020–2024).

Year	Buddhism	Taoism	Catholicism	Christianity	Islam
2020	0.3682	0.3782	0.3833	0.4109	0.3579
2021	0.3742	0.3850	0.3904	0.4248	0.3621
2022	0.3682	0.3935	0.3865	0.4202	0.3511
2023	0.3711	0.3946	0.4056	0.4288	0.3690
2024	0.3922	0.3912	0.4088	0.4058	0.3599
Mean	0.3747	0.3885	0.3949	0.4181	0.3600

The ANOVA results for the CV values of the religions showed an F-value of 28.7539 and a P-value of 4.84 × 10⁻⁸. The high F-value and extremely low P-value indicate statistically significant differences in regional variation between the religions, suggesting these differences are systematic rather than random. Such variation may stem from regional cultural influences, population distribution, and the historical presence of each religion in different regions. These findings highlight the importance of considering regional contexts when analysing religious trends in China. They also provide practical implications for policymakers and faith-based organizations, who can use these insights to develop region-specific advocacy, engagement, and resource allocation strategies that address the diverse needs of believers across different regions.

#### Spatial autocorrelation analysis

Global spatial autocorrelation in public attention to China’s five major religions was analysed using GeoDa software (Eqs. 4–6), examining patterns across all 31 regions during the 2020–2024 study period. As shown in Table 4, the Global Moran’s *I* values for all five religions remain positive, albeit relatively low, indicating weak but consistent global spatial clustering. Notably, an overall upward trend suggests that public attention is becoming increasingly spatially aggregated over time. This trend implies that the distribution of religious interest is progressively shaped by geographic space rather than occurring randomly. The internet, as a key medium of information dissemination, may reinforce these spatial clustering effects. Such spatial concentration has practical implications for religious dissemination and governance. Religious organisations and policymakers can leverage these patterns to promote targeted religious education, cultural exchange, and faith-based outreach within clustered regions.

Among the five religions, Catholicism displays the highest mean Global Moran’s I (0.2609), reflecting stronger spatial clustering, while Islam shows the lowest (0.0353), indicating a more dispersed attention pattern. These differences suggest the need for religion-specific digital strategies. For Catholicism, efforts could focus on deepening engagement within established clusters. In contrast, the spatial dispersion of Islam indicates a need to expand online outreach nationally, overcoming regional fragmentation to enhance visibility and influence.

**Table 4:** Global Spatial correlation of public attention to china’s five major religions (2020–2024).

Category		2020	2021	2022	2023	2024	Mean
Buddhism	I value	0.0900	0.1234	0.1423	0.1460	0.1836	0.1371
	Z-Score	1.0463	1.3380	1.4949	1.5081	1.8295	1.4434
Taoism	I value	0.1248	0.1504	0.1716	0.1474	0.1585	0.1505
	Z-Score	1.3432	1.5618	1.7386	1.5371	1.6362	1.5634
Catholicism	I value	0.2319	0.2639	0.2547	0.2689	0.2853	0.2609
	Z-Score	2.2503	2.5136	2.4265	2.5690	2.6943	2.4907
Christianity	I value	0.2048	0.2520	0.2486	0.2208	0.2540	0.2360
	Z-Score	2.0132	2.4172	2.3892	2.1606	2.4372	2.2835
Islam	I value	0.0127	0.0473	0.0377	0.0306	0.0481	0.0353
	Z-Score	0.3830	0.6945	0.6024	0.5482	0.6993	0.5855

A significance test of Z-scores revealed that Catholicism and Christianity consistently exceeded the 1.96 threshold across all five years (2020–2024), indicating statistically significant spatial autocorrelation. In contrast, Buddhism and Taoism showed moderate spatial clustering, with Z-scores fluctuating around or slightly below the threshold in certain years. Islam, by comparison, exhibited the weakest spatial autocorrelation, with Z-scores well below 1.96 throughout the period.

The ANOVA analysis yielded an F-value of 78.6520 and a P-value of 6.05 × 10⁻¹², confirming statistically significant differences in spatial clustering across the five religions. These variations may be shaped by regional demographics, cultural traditions, and the historical distribution of each faith. For religious organisations and policymakers, this highlights the need for spatially differentiated strategies in education, promotion, and engagement efforts.

Through the analysis of Global Moran’s *I*, this study reveals the spatial agglomeration pattern of Chinese religious public concern. It highlights the importance of incorporating spatial factors into the study of religious trends, as religions exhibit different geographical features that require tailored communication and policy approaches. These findings demonstrate a profound understanding of how religious influences spread in the digital era and provide practical guidance for strengthening religious governance and promoting harmony. For instance, policymakers may prioritise areas with consistently high levels of religious engagement when designing cultural preservation initiatives and policy interventions, ensuring alignment with local religious and social dynamics.

However, Moran’s *I* does not identify specific local clustering patterns. Therefore, Eq. (5) was used to conduct Local Moran’s *I* analysis to detect the local spatial heterogeneity of public attention. According to the results of each region, the spatial clustering types are classified, as shown in Table 5.

**Table 5:** Local Spatial correlation types of public attention to china’s five major religions (2020–2024).

Year		H–H	L–L	H–L	L–H
Buddhism	2020	Shandong, Anhui, Jiangsu, Fujian	Xinjiang, Gansu	Sichuan	Jiangxi, Hainan
	2021	Shandong, Anhui, Jiangsu, Fujian	Xinjiang, Gansu, Qinghai	Sichuan	Jiangxi, Hainan
	2022	Shandong, Anhui, Jiangsu, Shanghai, Fujian	Xinjiang, Gansu, Qinghai	Sichuan	Jiangxi, Hainan
	2023	Shandong, Anhui, Jiangsu, Shanghai, Fujian	Xinjiang, Gansu	Sichuan	Jiangxi, Hainan
	2024	Shandong, Anhui, Jiangsu, Shanghai, Fujian	Xinjiang, Gansu, Qinghai	Sichuan	Jiangxi, Hainan
Taoism	2020	Shandong, Jiangsu, Fujian	Xinjiang, Gansu, Qinghai	Sichuan	Anhui, Jiangxi, Hainan
	2021	Shandong, Jiangsu, Fujian	Xinjiang, Gansu, Qinghai	Sichuan	Anhui, Jiangxi, Hainan
	2022	Shandong, Anhui, Jiangsu, Fujian	Xinjiang, Gansu, Qinghai	Sichuan	Jiangxi, Hainan
	2023	Shandong, Anhui, Jiangsu, Fujian	Xinjiang, Gansu	Sichuan	Jiangxi, Hainan
	2024	Shandong, Anhui, Jiangsu, Fujian	Xinjiang, Gansu, Qinghai	Sichuan	Jiangxi, Hainan
Catholicism	2020	Shandong, Henan, Jiangsu, Shanghai, Fujian	Xinjiang, Gansu, Qinghai	Sichuan	Anhui, Jiangxi, Tianjin, Hainan
	2021	Shandong, Henan, Jiangsu, Shanghai, Fujian	Xinjiang, Gansu, Qinghai	Sichuan	Anhui, Jiangxi, Tianjin, Hainan
	2022	Shandong, Henan, Jiangsu, Shanghai, Fujian	Xinjiang, Gansu, Qinghai	Sichuan	Anhui, Jiangxi, Tianjin, Hainan
	2023	Shandong, Henan, Jiangsu, Fujian	Xinjiang, Gansu, Qinghai	Sichuan	Anhui, Jiangxi, Tianjin, Hainan
	2024	Shandong, Henan, Jiangsu, Shanghai, Fujian	Xinjiang, Gansu, Qinghai	Sichuan	Anhui, Jiangxi, Tianjin, Hainan
Christianity	2020	Shandong, Anhui, Jiangsu, Shanghai, Fujian	Xinjiang, Gansu, Qinghai	Sichuan	Jiangxi, Hainan
	2021	Shandong, Anhui, Jiangsu, Shanghai, Fujian	Xinjiang, Gansu, Qinghai	Sichuan	Jiangxi, Hainan
	2022	Shandong, Anhui, Jiangsu, Shanghai, Fujian	Xinjiang, Gansu, Qinghai	Sichuan	Jiangxi, Hainan
	2023	Shandong, Anhui, Jiangsu, Shanghai, Fujian	Xinjiang, Gansu, Qinghai	Sichuan	Jiangxi, Hainan
	2024	Shandong, Anhui, Jiangsu, Shanghai, Fujian	Xinjiang, Gansu, Qinghai	Sichuan	Jiangxi, Hainan
Islam	2020	Anhui, Fujian	Xinjiang	Sichuan	Jiangxi, Hainan
	2021	Anhui, Jiangsu, Shanghai, Fujian	Xinjiang	Sichuan	Jiangxi, Hainan
	2022	Shandong, Anhui, Jiangsu, Shanghai, Fujian	Xinjiang	Sichuan	Jiangxi, Hainan
	2023	Shandong, Anhui, Jiangsu, Shanghai, Fujian	Xinjiang	Sichuan	Jiangxi, Hainan
	2024	Shandong, Anhui, Jiangsu, Shanghai, Fujian	Xinjiang	Sichuan	Jiangxi, Hainan

**Fig. 3:**
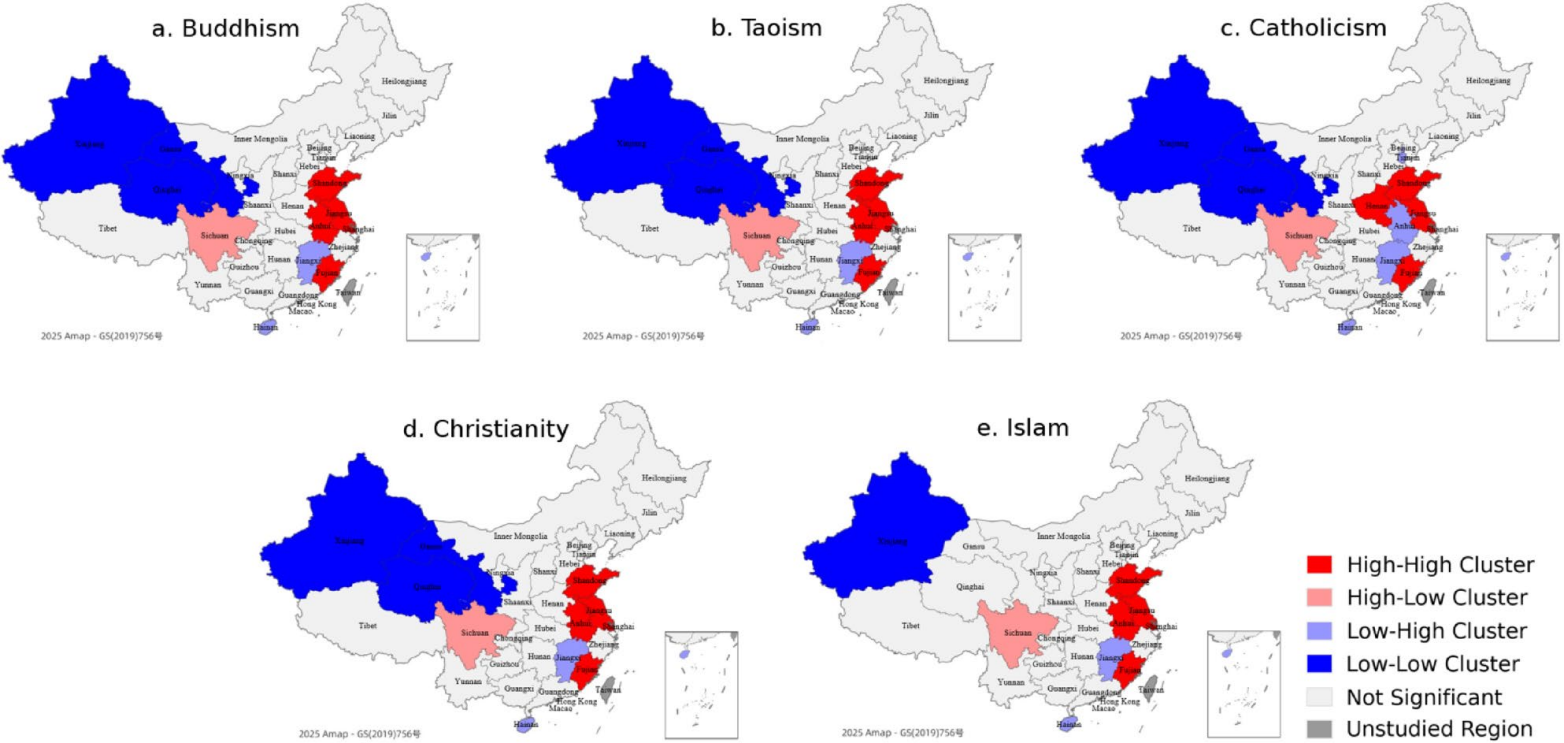
Local spatial correlation types of public attention to China’s five major religions in 2024.

Utilising GeoDa software to visualise the spatial distribution of public attention to China’s five major religions across 31 regions in 2024. The resulting local spatial autocorrelation map is presented in Fig. 3.

The results (Fig. 3; Table 5) reveal notable spatial clustering patterns, categorised into four types: H–H, L–L, H–L, and L–H.

(1) Positive correlation regions (H–H and L–L)

Regions with significant positive correlation include Shandong, Jiangsu, Shanghai, and Fujian (H–H), and Xinjiang, Gansu, Qinghai (L–L), commonly appearing across Buddhism, Taoism, Catholicism, and Christianity. These areas represent 25.81% of the total, indicating strong spatial clustering. Eastern provinces show high public attention due to advanced economies, internet access, and education levels, while western L–L regions reflect consistently low attention. Religious organisations may enhance content delivery in H–H areas and improve infrastructure and outreach in L–L areas.

(2) Negative correlation regions (H–L and L–H)

H–L and L–H types appear in regions such as Sichuan, Jiangxi, Anhui, Tianjin, and Hainan, accounting for around 9.68–16.13%. These regions reflect spatial heterogeneity in religious attention, possibly driven by economic, cultural, or policy differences. Targeted communication strategies are needed to address transmission barriers and align religious content with local contexts.

(3) Areas with no significant autocorrelation

Over 50% of regions show no significant spatial autocorrelation, showing random or independent distribution of public attention. In such areas, religious influence is likely shaped by local factors such as demographic structure, cultural diversity, and economic conditions. Flexible, locally adapted outreach strategies are essential.

(4) Inter-religious spatial clustering differences

While Buddhism, Taoism, Catholicism, and Christianity exhibit relatively strong clustering, Islam shows weaker spatial correlation and fewer positive clustering regions. This spatial inconsistency is particularly evident in regions such as Xinjiang, which is demographically known for its large Muslim population but is classified as a L–L cluster in our results. A likely explanation lies in Baidu Index data—it captures online search behaviour rather than actual religious affiliation. Moreover, Baidu Index attributes search activity to users’ IP-based locations^73^ which may not reflect their actual place of residence. This can result in a mismatch between search flow and population flow. In regions with limited internet access, low levels of digital engagement, or restricted media environments, public attention may be underrepresented despite a high concentration of believers. Therefore, the spatial patterns observed for religions in this study reflect public attention rather than demographic prevalence and should be interpreted accordingly.

While Buddhism and Taoism appear spatially similar, subtle differences emerge upon closer inspection. For example, Table 5 shows that Shanghai is classified as an H–H cluster for Buddhism but not for Taoism. These distinctions, while minor, suggest that public attention toward these two traditional religions is not entirely identical. Such differences may stem from varying cultural expressions, media presence, or the contemporary relevance of each religion in different regions.

Overall, the spatial variation reflects differences in religious origins, modes of dissemination, cultural adaptability, and public perception. Religions with deeper historical roots and broader cultural integration tend to exhibit stronger spatial clustering in public attention, while religions with highly localised demographics may require broader communication strategies.

(5) Temporal Stability and Change

Spatial patterns of public attention show overall stability over time, with consistent negative correlation regions and minor variations in positive clusters. This suggests that while spatial dissemination is persistent, it is also shaped by dynamic factors such as socioeconomic shifts and policy adjustments. Long-term planning for religious governance should be based on these stable patterns while allowing timely responses to emerging changes.

**Fig. 4:**
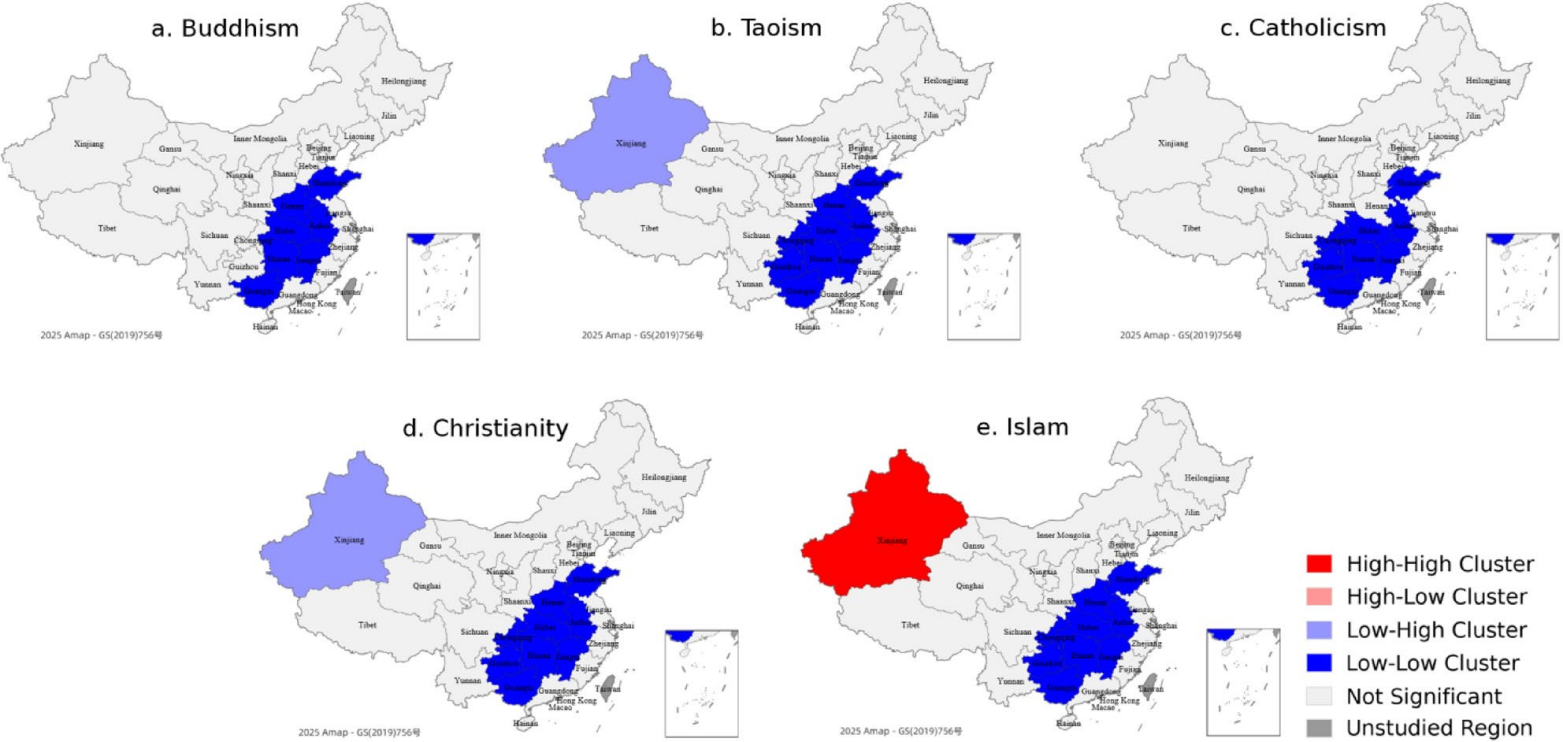
Normalised local spatial correlation types of public attention to China’s five major religions in 2024.

Recognising that relying on non-normalised Baidu Index data may introduce bias by favouring more populous regions, this study conducted a supplementary spatial autocorrelation analysis using Baidu Index data normalised by provincial population size. The provincial population data were sourced from the National Bureau of Statistics. The normalised LISA maps are shown in Fig. 4. Compared with the non-normalised LISA maps (Fig. 3), the spatial clustering patterns based on the normalised data reveal several notable changes.

First, the previously dominant H–H clusters observed in populous provinces such as Shandong, Henan, Hunan, and Jiangsu either disappear or shift to L–L clusters in the normalised maps. This suggests that their dominant levels of public attention may have been driven by large population sizes rather than by genuine per capita interest in religious topics.

Second, a significant shift is observed in Xinjiang, which was previously classified as a L–L cluster for all religions, but now appears as an H–H cluster for Islam after population adjustment. This result aligns more closely with the known demographic concentration of Muslim populations in the region and highlights how population-adjusted metrics reflect demographic realities.

Third, western provinces such as Qinghai and Gansu, which were previously classified as L–L clusters, now appear as not significant in the normalised maps. This shift suggests that their earlier clustering was driven more by low absolute search volumes than by genuinely low per capita attention. After normalisation, their per capita attention levels are no longer significantly lower than those of neighbouring regions, indicating a more neutral spatial distribution.

These changes confirm that population size exerts a structural influence on public attention data and that normalisation provides a more balanced basis for spatial comparison. While detailed religion-specific population data remain unavailable, this population-based adjustment serves as a reasonable proxy and enhances the validity of spatial analysis. Even so, the normalised results help mitigate population-driven bias and improve the interpretive credibility of digital public attention to religion.

### Public attention forecasting model

#### Stationarity test of the time series

The dataset was divided into training (2020–2023) and validation (2024) periods to assess the forecasting model’s accuracy. Using Buddhism as an example, a forecasting model was constructed based on its public attention time series. As presented in Table 6, the ADF stationarity test results for the original time series (*p* > 0.05) indicate non-stationarity. Following first-order differencing, the test statistic becomes significant (*p* < 0.05), rejecting the null hypothesis of a unit root and confirming stationarity in the transformed series.

**Table 6:** Time series ADF stationarity test of buddhism.

Series	Statistics	Critical value5% level	*p*-value
Original time series	−2.30	−2.93	0.1724
First-order differenced series	−10.22	−2.93	5.26 × 10^−18^

**Fig. 5:**
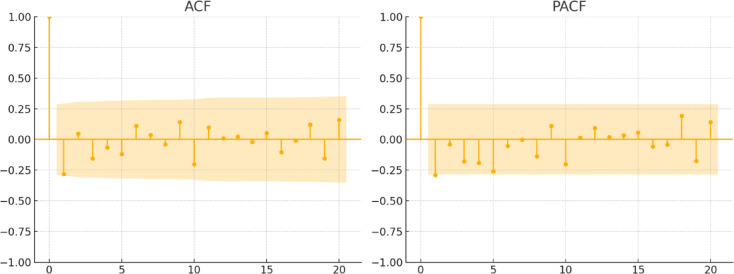
ACF and PACF of the first-order differenced series.

Figure 5 presents the autocorrelation function (ACF) and partial autocorrelation function (PACF) of the first-order differenced series. The absence of clear seasonal patterns in the plots indicates that the data meet the conditions for constructing a SARIMA model.

#### Model construction and parameter testing

Preliminary analysis of the Buddhism public attention time series indicates that the series meets the requirements for SARIMA modelling after first-order non-seasonal differencing. Accordingly, the differencing order (*d*) was set to 1, and the seasonal period (*s*) to 12.

MAPE reflects the relative deviation between forecasted and actual values, and this study selects the optimal model based on the minimum MAPE. A grid search was performed across all possible parameter combinations to determine the optimal model. The SARIMA (1,0,2)×(1,0,0)_12_ model achieved the best forecasting performance for Buddhism.

Model residuals were assessed using the Ljung–Box Q test (lag = 12), yielding a p-value of 0.8858, which is well above the 0.05 threshold, confirming that the residuals constitute a white noise process. This confirms that the SARIMA model has effectively captured the structural components of the original series and is suitable for forecasting.

Similarly, optimal SARIMA models were identified for Taoism, Catholicism, Christianity, and Islam, all of which passed the Ljung–Box test, validating their adequacy. The final optimal models and corresponding Ljung-Box/Q test results for the five major religions are presented in Table 7.

**Table 7:** Optimal SARIMA models and parameter test results.

Religion	Optimal model	Ljung-Box *p*-value
Buddhism	SARIMA (1,0,2)×(1,0,0)_12_	0.8858
Taoism	SARIMA (0,1,0)×(0,1,1)_12_	0.1232
Catholicism	SARIMA (0,2,1)×(0,1,1)_12_	0.9472
Christianity	SARIMA (0,1,0)×(1,1,1)_12_	0.6680
Islam	SARIMA (1,2,1)×(1,1,1)_12_	0.4648

#### Model fitting and forecasting

Model forecasting performance was assessed using three key evaluation metrics: MAPE, RMSE, and DA. As shown in Table 8, the forecasting fitting errors for all religions are below 10%, indicating a generally good model fit. Catholicism demonstrated superior predictive performance among all models, with a MAPE of 4.44% and an RMSE of 58.49, reflecting the lowest forecasting error and strong stability in volatility control. The Buddhism model achieved the highest DA at 72.73%, indicating superior capability in capturing directional trends.

By contrast, the Christianity model showed slightly weaker results in both RMSE and DA, suggesting limited effectiveness in handling trend fluctuations. Overall, except for the Christianity model, the other four models showed satisfactory performance in both fitting accuracy and trend detection, demonstrating their practical value for short-term forecasting.

**Table 8:** Evaluation metrics of model fitting Accuracy.

Religion	MAPE	RMSE	DA
Buddhism	6.29%	143.96	72.73%
Taoism	6.69%	221.03	63.64%
Catholicism	4.44%	58.49	63.64%
Christianity	7.87%	253.25	45.45%
Islam	8.86%	238.62	63.64%

**Fig. 6:**
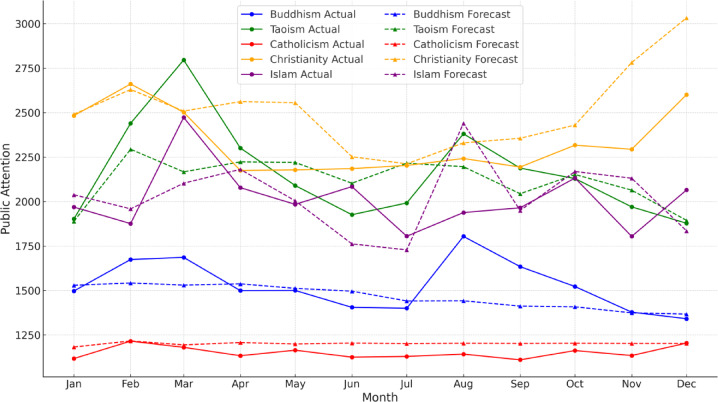
Forecasting result of SARIMA model of Religions in 2024.

The SARIMA forecasting results are illustrated in Fig. 6. The forecasted trend relatively aligns with the actual data, effectively capturing the linear fluctuation patterns. Christianity shows the highest and most steadily increasing attention, especially with a sharp rise in December, though forecasts slightly overestimate in the final months. Taoism exhibits clear seasonal spikes in March and August, which the model fails to anticipate. Buddhism remains relatively stable but shows a major unexpected surge in August, underpredicted by the model. Islam also has a noticeable peak in March that is not captured by the forecast, likely linked to religious observances. Catholicism maintains the lowest and most stable attention, with high consistency between actual and forecasted values.

Overall, the findings validate the applicability of the SARIMA model across all religions. While the model effectively captures general seasonal trends—particularly for religions like Catholicism with relatively stable attention—it underperforms in identifying event-driven spikes associated with Taoism, Islam, and Buddhism. This highlights the need to incorporate external event variables or enhance the model’s temporal sensitivity to improve forecasting accuracy.

## Discussion

### Interpretation of key findings

This study aims to explore the spatiotemporal distribution patterns and forecasting model of public attention to China’s five major religions, using GIS technology and Baidu Index data. Based on the research objectives and research questions, the key findings are further discussed as follows:

#### Time characteristics

By analysing the temporal distribution characteristics of public attention across China’s five major religious, this study identifies several key patterns and trends.

Annual fluctuations in public attention are relatively minor, with no significant differences in AVI across religions. This consistency suggests that, overall, online religious interest remains stable year-on-year. Nevertheless, even subtle variations can serve as useful indicators for religious organisations to monitor engagement levels and adapt their activities accordingly^54,92^.

Public attention demonstrates strong seasonal trends, often influenced by religious festivals and cultural observances. For example, Christianity peaks in December due to Christmas^93^ while Buddhism’s interest increases in May, possibly related to Buddha’s birthday or related events^94^. These trends are closely tied to religious calendars, underscoring the role of tradition in shaping digital engagement. Recognizing seasonal dynamics enables organizations to schedule their outreach efforts more effectively^95^ such as releasing targeted content during key events to maximize visibility and engagement^96^.

Different religions show different degrees of online visibility. Christianity consistently ranks high while Catholicism shows relatively low, suggesting differences in digital strategies or approaches to community engagement. This trend calls for further investigation into how faith-based institutions can leverage digital platforms for outreach, as increasingly sophisticated digital strategies can generate sustained or enhanced visibility^56^.

In summary, while annual changes remain stable, seasonal and event-based fluctuations are significant^50^. Differences in digital visibility among religions also highlight the need for a differentiated strategy. For faith-based organizations, aligning digital outreach with seasonal trends and monitoring annual engagement patterns is critical to staying relevant in an increasingly digital society. Moreover, these time patterns may reflect broader global dynamics. The insights from this study provide a useful reference for cross-cultural research and may help to shed light on common temporal trends in religious concerns worldwide.

#### Spatial characteristics

This study examined the regional differences, centralized distribution and spatial aggregation of public attention to China’s five major religious, and revealed the spatial pattern and regional differences of the five major religious public attention in China.

The CV values of all religions were greater than 0.3, indicating a moderate degree of difference in spatial variation. These differences are not random, and probably reflect differences in regional culture, religious customs and population composition^97^. The uneven geographical distribution of religious believers and local cultural preferences may lead to the level of concern in specific regions. For policy makers and religious organizations, the recognition of these regional differences is essential for the development of targeted strategies and more effective allocation of resources.

The Global Moran’s *I* values of the five religions are all positive, suggesting that the public attention in religious is spatially clustering rather than randomly distributed^56^. Catholicism shows the highest average Moran’s *I*, indicating a strong aggregation, which may be due to the establishment of perfect faith communities in coastal cities such as Shanghai, Fujian and Guangdong^98^. The spatial concentration of Islam is the lowest, mainly concentrated in the northwest ethnic minorities area, which is geographically dispersed and has a low population density^99^.

Local Moran’s *I* analysis provides further insights. The H-H cluster is mainly in the eastern provinces, which is related to a high degree of public concern, which can be attributed to a stronger economy, better Internet infrastructure and higher education level^23^. On the contrary, L–L clusters are concentrated in western regions, such as Xinjiang, Gansu and Qinghai. The geographical isolation and cultural differences in these regions may limit online participation. Sichuan, Anhui, Jiangxi, Hainan and Tianjin present L–H and H–L patterns, reflecting the spatial heterogeneity of religious public attention. This may stem from communication barriers, policy inconsistencies, or cultural divergence between regions. In addition, more than half of the provinces show no significant spatial autocorrelation, indicating the random distribution of public attention. These regions need further investigation to determine the driving factors of local religious participation and provide information for the development of more nuanced and environmentally sensitive outreach strategies.

The results show that public attention is usually geographically concentrated, which supports the theory of spatial diffusion and religious clustering^100,101^. This has practical significance for the government and religious organizations. In the H-H regions, policy intervention can focus on consolidating religious and cultural activities. In the L–L regions, improving Internet infrastructure and promoting cultural development may help balance communication. For L–H and H–L regions, efforts should be made to overcome communication barriers and provide content consistent with local cultural background to improve participation.

The spatial distribution patterns also vary between religions. Traditional religions such as Buddhism and Taoism show strong regional concentration, which may be due to their profound historical roots and cultural integration. In contrast, Islam is more scattered in space, reflecting the concentration of its population in specific areas. These differences highlight the connection between religious communication and regional culture^56^. To promote religious exchanges and mutual understanding among regions is conducive to promoting the harmonious integration of religions^57^.

In conclusion, public attention to China’s five major religions generally presents moderate spatial differences and relatively balanced regional distribution. Generally, it presents a pattern of east–west clustering with H–H cluster concentrated in the East and L–L cluster concentrated in the West. These spatial patterns reflect the influence of history, culture and social economy on religious distribution, and highlight the value of incorporating a spatial perspective in the analysis of religious communication in the digital era. Moreover, the spatial trends identified in this study may have global relevance, providing new insights for the development of cultural and regional adaptive strategies in comparative research, international dialogue and religious governance and communication.

Despite the meaningful patterns found in this study, it is important to note that the Baidu Index data used in this study primarily reflect public attention rather than actual number of religious believers^20^. Due to the lack of reliable and consistent data on religious populations across provinces and over time, we were unable to perform religion-specific demographic normalisation.

To partly address this limitation and explore the potential influence of population size on spatial clustering, a supplementary analysis was conducted using Baidu Index values normalised by provincial population size. The results (see Fig. 4) show clear differences compared to the unromanised maps. For example, populous provinces such as Shandong, Henan, and Jiangsu, which were previously identified as H–H clusters, lost their dominant status or shifted to L–L clusters after normalisation. In contrast, Xinjiang changed from a L–L cluster to a H–H cluster for Islam, which better reflects its demographic background. Qinghai and Gansu also moved from L–L clusters to non-significant status, suggesting that their earlier low absolute search volumes were partly due to low population.

The findings suggest that spatial clustering is shaped by population size^23^ and population adjustment helps reveal more balanced patterns of public attention. Although this method is not a perfect substitute for religion-specific data, it helps reduce bias and gives a more balanced picture. Accordingly, the spatial patterns identified in this study should be understood as reflections of online interest rather than direct representations of religious population prevalence. This distinction is crucial for policymakers and scholars seeking to apply these insights in digital governance, cultural communication, or comparative studies.

Moreover, Baidu Index determines user location based on IP addresses, which may not reflect the user’s actual place of residence. This can lead to a mismatch between search flow and population flow^102^. As discussed in Sect. “Spatial autocorrelation analysis”, such mismatch may partly explain anomalies like the initial Low–Low clustering of Xinjiang, despite its large Muslim population. These findings reinforce that the spatial patterns observed reflect digital attention rather than actual religious demographics, and should be interpreted with this limitation in mind^20^.

#### Forecasting model

Through a systematic analysis of the temporal distribution pattern of public attention to China’s five major religions, it is found that the monthly and annual fluctuation is generally stable. This provides a theoretical basis for the application of linear time series models such as SARIMA. Taking Buddhism as an example, the selected optimal model SARIMA (1,1,2)×(1,0,0)_12_ performs well in the residual stability, white noise diagnosis, prediction accuracy and confidence interval coverage. The model effectively catches the time dynamic of public attention to Buddhist, and provides a strong forecasting and explanatory power.

The effectiveness of the SARIMA model supports previous findings, demonstrating that public attention to most religions shows relatively weak fluctuations on a monthly and annual level, and its model shows a high regularity^92^. The stability captured by the model is consistent with the continuity of digital religion participation^103^ indicating that Buddhism and other major religions remain consistent online throughout the year. This phenomenon may be due to the long-term cultural significance of religion and the growing maturity of digital communication channels^104^.

Furthermore, by comparing the output of the model with the holiday effect of the existing records, this study found that the increase of Buddhist attention observed in May coincided with religious festivals such as the birthday of Buddha. Although the seasonal effects do not dominate the trend, they seem to be embedded in the seasonal autoregressive or cyclical components of the model, so that they can indirectly reflect the fluctuation. This discovery highlights the dynamic interaction between religious ceremonies and digital attention, and provides empirical support for religious organizations to strengthen their outreach strategies during key festivals^105,106^.

In summary, SARIMA model can not only adapt to the overall temporal pattern, but also be sensitive to cultural and seasonal variations. It provides a powerful analysis framework for digital religion research and a quantitative basis for faith-based institutions to optimize participation time. Moreover, this approach has a broader global relevance, which helps to better understand the temporal dynamics and behaviour patterns of religious digital communication in different social and cultural backgrounds.

### Implications

This study has an important contribution in theory, practice and methods, advancing the field of religious studies.

Theoretically, this study applies GIS technology, spatiotemporal indicators and forecasting model to investigate the public attention to China’s five major religions. The introduction of quantitative spatiotemporal method to religious research provides a new research perspective and analysis framework for the international academic community. The research promotes the interdisciplinary integration of religious research, geography and sociology, and supports further academic dialogue on transnational religious behaviour, interfaith exchanges and the social role of religion. Moreover, the application of spatial autocorrelation analysis provides empirical support for spatial clustering and diffusion, and expands their relevance in digital religion research.

Practically, the study reveals the spatiotemporal distribution patterns of public attention to China’s five major religions, providing insights for the evolution of religious landscape in the digital era. These findings enhance the understanding of public religious interests, and provide information for policy-making and management by providing data-driven decision support for relevant institutions. The results also emphasize the social function of religion and its potential to promote social harmony and cultural cohesion. Although based on China’s data, the methods and results of this study are related to a broader global background, which can provide a reference for cross-cultural and cross-regional studies of religious communication and governance in an increasingly interconnected world.

Methodologically, this study demonstrates the application value of spatiotemporal indicators, spatial autocorrelation analysis and forecasting technology in religious research. These tools are not only suitable for religious research, but also for broader social science research. Using GIS to visualize spatiotemporal patterns provides an intuitive and effective way to present complex data, which improves the accessibility and communication of research results. This study provides a valuable method reference for the future interdisciplinary and data-driven research in the international academic community.

### Limitations and recommendations

However, there are some limitations concerning data sources, analysis methods, and exploration of influencing factors. First, the analysis is only based on Baidu index data, which reflects online search behaviour rather than actual religious adherence. While it provides insight into digital public attention, it may not fully represent the broader religious concerns of China’s population. Regions with many religious believers but limited internet access may show low levels of online attention. In contrast, areas with better internet coverage and more media exposure may appear to have higher attention, even if their religious population is smaller. This limitation introduces potential sampling bias and highlights the need to interpret Baidu-based findings as indicators of digital interest rather than demographic prevalence. Second, although widely used, the temporal and space indicators used in this study are more effective than capturing short-term fluctuations in determining the overall trend, which may limit its sensitivity and accuracy. Additionally, the spatial similarity observed across different religions may partly stem from the overlapping influence of digital infrastructure, economic development, and urbanisation, which affect search behaviour more than religious identity. Third, the study did not comprehensively consider the social, political and cultural factors that affect religious public attention. Only relying on Baidu index data limits the ability to explore these factors in depth.

Future research should expand data sources and improve methods. Integrating data from other search engines, social media and online resources will produce more representative datasets^107^ while real-time data collection could enhance the timeliness and accuracy of findings^108^. Cross-validating online attention data with offline religious population statistics, field survey data, or government census where available would further improve validity and reliability. Methodologically, using finer temporal scales, such as weekly or daily data, and advanced spatial techniques, including spatial regression and geographic detectors, may reveal deeper regional patterns^109,110^. For forecasting, integrating big data technologies and deep learning models could significantly improve accuracy and adaptability to changing trends^96,111,112^. Furthermore, combining qualitative and quantitative methods, such as multi-level regression or causal model, will be able to more comprehensively analyse the factors that lead to the temporal and spatial changes of religious public attention^113^ thereby strengthening both theoretical understanding and policy relevance.

## Conclusion

This study explored the spatiotemporal distribution and forecasting patterns of public attention to China’s five major religions using Baidu Index data from 2020 to 2024. The results revealed relatively stable annual trends with periodic seasonal fluctuations, particularly around religious festivals and significant events. Spatially, public attention exhibited moderate regional variation and a distinct east–west clustering pattern. Christianity attracted the highest online attention, while Catholicism showed the most stability with the lowest variation. The application of the SARIMA model demonstrated effective performance in capturing temporal dynamics, achieving high forecasting accuracy for most religions, especially for Catholicism and Buddhism. However, event-driven attention surges posed forecasting challenges, suggesting areas for further improvement in temporal modelling.

This research offers significant theoretical and practical contributions. The integration of GIS, spatial autocorrelation analysis, and SARIMA forecasting models into the study of religion provides an innovative interdisciplinary framework that bridges religious studies, sociology, and geography. By visualising and predicting public religious attention across time and space, the study provides new insights into how digital interest in religion is shaped by socio-cultural and regional factors. These findings offer actionable implications for faith-based organisations and policymakers to enhance digital outreach, optimise content timing, and develop region-specific engagement strategies. Although grounded in the Chinese context, the methodological approach and insights are globally applicable to the study of religious communication and cultural diffusion in the digital age.

Nevertheless, the study has certain limitations. The reliance on Baidu Index data reflects only online search behaviour rather than actual religious participation, potentially leading to bias in regions with poor internet access. The analysis did not account for underlying social, political, or cultural variables that influence attention patterns, limiting explanatory depth. Future research should consider combining online data with offline indicators such as census statistics and survey data to better capture the real-world religious landscape. Methodologically, integrating multi-source data, finer time granularity (e.g., weekly), spatial regression models, and deep learning techniques could enhance the model’s forecasting power and sensitivity. Additionally, incorporating qualitative insights may help unravel the contextual drivers behind observed spatial and temporal patterns, leading to more comprehensive and policy-relevant findings.

## Supplementary Information

Below is the link to the electronic supplementary material.

Supplementary Material 1

## Data Availability

The data underlying this study are available at https://index.baidu.com (Baidu Index).
